# Construction and Validation of a Reliable Six-Gene Prognostic Signature Based on the TP53 Alteration for Hepatocellular Carcinoma

**DOI:** 10.3389/fonc.2021.618976

**Published:** 2021-06-10

**Authors:** Junyu Huo, Liqun Wu, Yunjin Zang

**Affiliations:** Liver Disease Center, The Affiliated Hospital of Qingdao University, Qingdao, China

**Keywords:** hepatocellular carcinoma, TP53, prognostic, signature, biomarker

## Abstract

**Background:**

The high mutation rate of TP53 in hepatocellular carcinoma (HCC) makes it an attractive potential therapeutic target. However, the mechanism by which TP53 mutation affects the prognosis of HCC is not fully understood.

**Material and Approach:**

This study downloaded a gene expression profile and clinical-related information from The Cancer Genome Atlas (TCGA) database and the international genome consortium (ICGC) database. We used Gene Set Enrichment Analysis (GSEA) to determine the difference in gene expression patterns between HCC samples with wild-type TP53 (n=258) and mutant TP53 (n=116) in the TCGA cohort. We screened prognosis-related genes by univariate Cox regression analysis and Kaplan–Meier (KM) survival analysis. We constructed a six-gene prognostic signature in the TCGA training group (n=184) by Lasso and multivariate Cox regression analysis. To assess the predictive capability and applicability of the signature in HCC, we conducted internal validation, external validation, integrated analysis and subgroup analysis.

**Results:**

A prognostic signature consisting of six genes (EIF2S1, SEC61A1, CDC42EP2, SRM, GRM8, and TBCD) showed good performance in predicting the prognosis of HCC. The area under the curve (AUC) values of the ROC curve of 1-, 2-, and 3-year survival of the model were all greater than 0.7 in each independent cohort (internal testing cohort, n = 181; TCGA cohort, n = 365; ICGC cohort, n = 229; whole cohort, n = 594; subgroup, n = 9). Importantly, by gene set variation analysis (GSVA) and the single sample gene set enrichment analysis (ssGSEA) method, we found three possible causes that may lead to poor prognosis of HCC: high proliferative activity, low metabolic activity and immunosuppression.

**Conclusion:**

Our study provides a reliable method for the prognostic risk assessment of HCC and has great potential for clinical transformation.

## Background

Hepatocellular carcinoma (HCC) is a major cause of cancer mortality due to its high incidence rate, high recurrence, and limited molecular targeted therapeutic options (1, 2). Thus, there is an urgent need to discover novel biomarkers and design novel therapeutic strategies for HCC.

TP53 mutations occur in almost every type of cancer at rates from 38%-50%. Additionally, it has been shown that TP53 mutations were more frequent in cancer patients with lower survival rates among all cancer types studied (3). Wild-type TP53 monitors abnormal activities in cells, senses cell pressure or damage, and prevents the proliferation of damaged cells (4). However, under TP53 mutation, cells exhibiting DNA damage are capable of escaping apoptosis and transforming into carcinoma cells. In addition, TP53 mutant proteins lose their wild-type function and accumulate in the nucleus, a significant hallmark of malignant tumors (5). A large sample study of 10225 cancer patients found that TP53 mutations were more frequent in cancer patients with lower survival rates among all 32 cancer types studied (6). Professor Donhower’s (6) study also found that in most TP53 mutant tumors, oncogene amplification increased, and tumor suppressor genes were deeply deleted. The expression patterns of RNA, miRNA and protein in TP53-mutated tumors are different from those in nonmutated tumors. The expression of cell cycle progression genes and proteins in TP53-mutated tumors is enhanced.

The TP53 mutation process is the most common mutation process in HCC. This gene is critical for maintaining the stable properties of the genome. Its loss of function can lead to a centrosome amplification process, aneuploid cell proliferation process and chromosome instability (7, 8). Some related reports showed that the TP53 mutation process was not only related to the prognosis of HCC (9) but also correlated with serum alpha-fetoprotein (AFP), clinical stage, vascular invasion, tumor differentiation and Child-Pugh grade (10–13). Therefore, TP53 mutation has a profound impact on the genomic structure, expression and clinical prospects of HCC. Understanding the effect of TP53 on the pathogenesis of HCC is critical to develop more effective treatments for HCC.

The more we learn about TP53, the more we can understand the basic biology of HCC and develop better treatments. In this study, we speculated that the change in the RNA expression pattern caused by TP53 mutation may be an important reason for the difference in prognosis. Based on this hypothesis, we have successfully developed an approach capable of predicting accurate HCC prognosis. Considering the accuracy and universal applicability of the model, the six genes included may be likely targets to treat HCC.

## Material and approach

### Research Object

The clinical and transcriptome (fragments per kilobase of transcript per million (FPKM)) data of 374 HCC samples were acquired from The Cancer Genome Atlas (TCGA-LIHC) website (https://portal.gdc.cancer.gov/projects/TCGA-LIHC). Among them, there were 116 cases of TP53 mutation and 258 cases of wild-type TP53, and a detailed list of TP53-mutated samples was obtained from the cBioPortal website (https://www.cbioportal.org/). A total of 365 cases had complete prognosis information. We acquired the gene expression profile and clinic-related data of the LIRI-JP dataset from the International Genome Consortium (ICGC) database (https://dcc.icgc.org/releases/current/Projects/LIRI-JP) for external validation (n=229). TCGA and ICGC were all based on the Illumina HiSeq platform. The work here fully complied with the publication guidelines of TCGA and ICGC. Our research was based on public databases; therefore, further approval from the local ethics committee was not needed. The detailed clinical information of the TCGA and ICGC cohorts is shown in **Table 1**.

**Table 1:** The clinical information of TCGA and ICGC cohort.

	TCGA	ICGC
Survival status		
Alive	235	187
Dead	130	42
Gender		
Female	108	61
Male	231	168
Age		
<=65	219	88
>65	120	141
Stage-TNM		
I-II	252	139
III-IV	87	90
Histologic grade		
G1-2	212	
G3-4	127	

### Gene Set Enrichment Analysis

To determine the difference in gene expression patterns between HCC samples with wild-type TP53 (n=258) and mutant TP53 (n=116) in the TCGA-LIHC cohort, we performed gene set enrichment analysis. The gene set file (h.all.v7.1.symbols.gmt) was used as the reference gene group. The threshold value was set as P < 0.05 and FDR < 0.25.

### Screening of Prognosis-Related Genes

We screened prognosis-related genes by univariate Cox regression analysis and Kaplan–Meier (KM) survival analysis. The genes with P values less than 0.05 obtained by the two methods were used for subsequent study.

### Construction and Validation of Prognostic Model

To improve the generalization ability of the established prognostic model, 365 HCC cases with complete prognostic information were randomly divided into two independent cohorts: a training cohort (n = 184) and a validation cohort (n = 181). The aforementioned process was implemented using the R package ‘Caret’. In the training cohort (n = 184), we used least absolute shrinkage and selection operator (LASSO) penalty Cox regression analysis to further select prognostic genes. The LASSO algorithm with penalty parameter tuning performed *via* 10-fold cross-validation was applied to exclude genes that may be highly correlated with other genes. We performed 1000 10-fold cross-validations of data sets and selected genes with more than 900 repetitions (14, 15). A subset of genes was determined by shrinking the regression coefficient using a penalty proportional to their size. The genes with nonzero regression coefficients were retained for subsequent multivariate Cox regression analyses (16, 17). Finally, the regression coefficient obtained by multivariate Cox regression analysis was multiplied by the expression level of each gene to construct a prognostic model. All subjects were divided into two risk groups according to the median risk of the training cohort, and individuals with a risk score higher or lower than the median risk were divided into high-risk and low-risk groups, respectively. The Kaplan-Meier (KM) survival curve and the receiver operating characteristic (ROC) curve were used to assess the predictive ability of the model. To evaluate whether the prognostic model is independent of the traditional clinical features, we conducted univariate and multivariate Cox regression analyses of the prognostic model and the traditional clinical features. For internal and external validation, the testing cohort (n = 181), TCGA cohort (n = 365), external validation cohort (ICGC, n = 229), whole cohort (all included samples, n = 594) and subgroup (n = 9) survival analysis were adopted to validate the predictive capability and applicability exhibited by the prognostication signal in HCC.

LASSO regression was implemented using the ‘glmnet’ R package. The survival curve was generated using Kaplan–Meier survival analysis and visualized using the R package ‘survminer’, and data were analyzed using the log-rank test. The ROC curves were drawn, and the corresponding AUC values were calculated using the R package ‘timeROC’.

### Gene Set Variation Analysis

We first used the ‘Limma’ R package to identify genes with differential expression (DEGs) between the high‐risk group and the low‐risk group in the TCGA and ICGC cohorts. Adjusted P value < 0.05 and absolute value of fold change (FC) >1 were suggested to indicate statistical significance. Then, we performed gene set variation analysis (GSVA) on the DEGs to identify prognosis-associated signaling pathways associated with the signature using the ‘GSVA’ R package. For GSVA, we chose Kyoto Encyclopedia of Genes and Genomes (KEGG) pathways as the reference and adj P value < 0.05 as the cutoff to screen significantly altered pathways.

### Correlation Analysis Between Signature and Immunity

We conducted single sample gene set enrichment analysis (ssGSEA, 29 immune-related gene sets representing immune cell type, function, and pathway) to quantify the activity or enrichment levels of immune cells, functions, or pathways in the high- and low-risk samples from TCGA and ICGC, respectively. The normalized enrichment score (NES) calculated from ssGSEA was calculated using the ‘GSVA’ R package. We used independent-samples t tests to explore the differences in immune infiltration levels and immune function between the high- and low-risk groups, and P < 0.05 was suggested to indicate statistical significance.

## Results

### Gene Set Enrichment Analysis of TP53 in the TCGA Database

**Figure 1:**
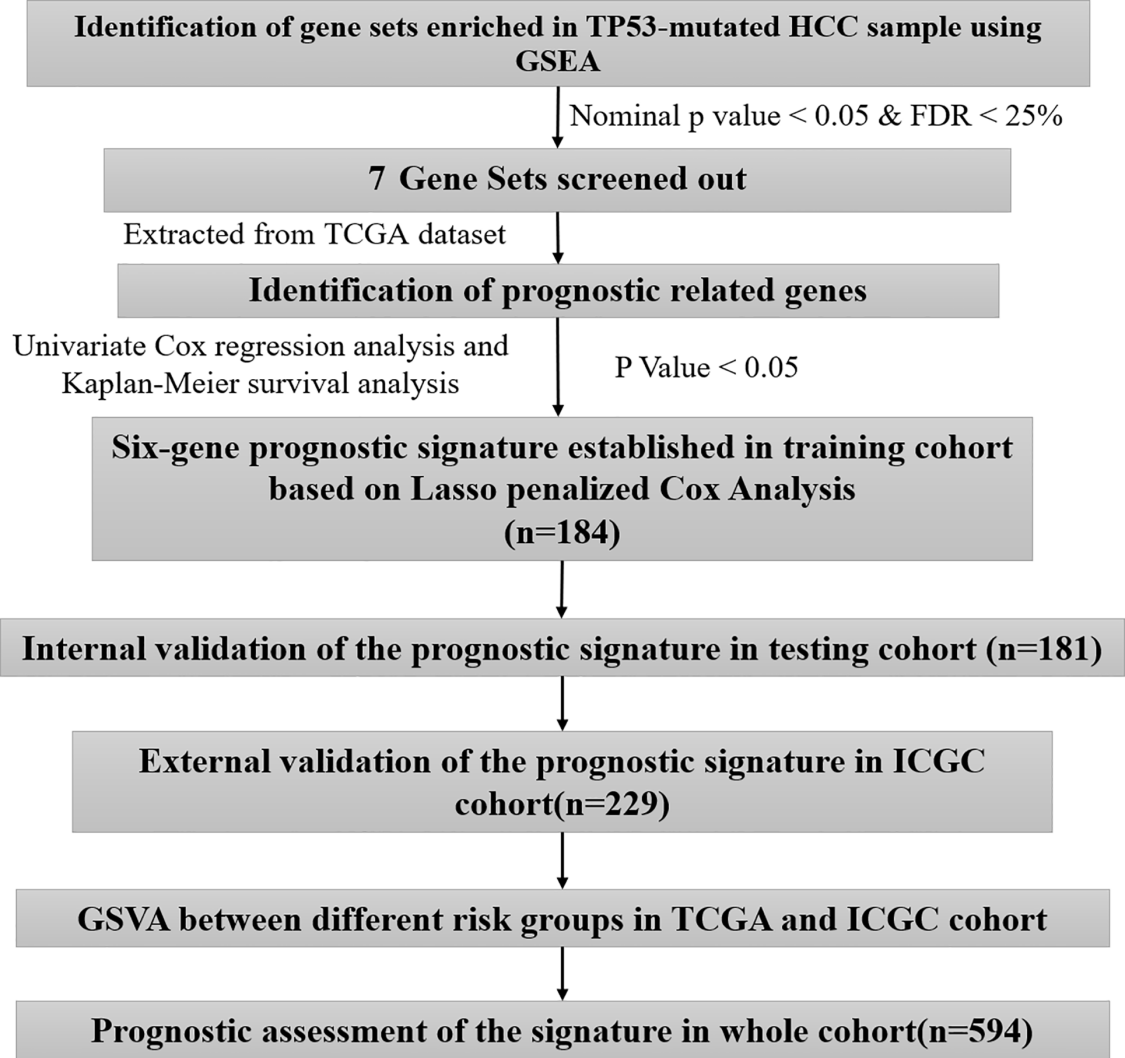
The workflow chart of this study.

**Figure 2:**
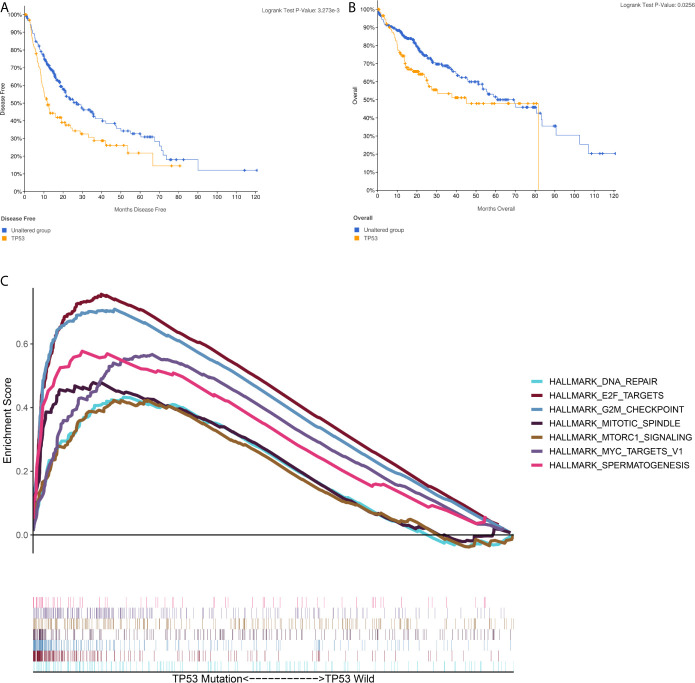
Gene Set Enrichment Analysis(GSEA) in TP53-mutated HCC sample **(A)** The Kaplan–Meier survival curve regarding the TP53 and disease free survival **(B)** The Kaplan–Meier survival curve regarding the TP53 and overall survival **(C)** The 7 gene sets identified by GSEA in TP53-mutated HCC sample.

A brief workflow for this study is shown in **Figure 1**. Compared to the unaltered group, the patients with TP53 mutation had both significantly poorer overall survival and disease-free survival (**Figures 2A, B**). We carried out GSEA of HCC samples from wild-type TP53 (n=258) and mutant TP53 (n = 116) samples. The results showed that 7 of the hallmark gene sets (n = 50) were significantly enriched in TP53-mutated HCC samples (**Table 2**, **Figure 2C**). We extracted 1048 genes from these 7 gene sets for subsequent analysis.

**Table 2:** The 7 gene sets identified by GSEA in TP53-mutated HCC sample.

NAME	SIZE	ES	NES	NOM p-val	FDR q-val
HALLMARK_E2F_TARGETS	184	0.75597	2.193104	0	0.001166
HALLMARK_G2M_CHECKPOINT	181	0.709121	2.109249	0.002	0.001891
HALLMARK_SPERMATOGENESIS	62	0.577112	2.012077	0.002101	0.007034
HALLMARK_MYC_TARGETS_V1	198	0.566704	1.822196	0.035052	0.037724
HALLMARK_MITOTIC_SPINDLE	183	0.478536	1.662072	0.038911	0.096263
HALLMARK_MTORC1_SIGNALING	191	0.423001	1.655123	0.041485	0.083437
HALLMARK_DNA_REPAIR	143	0.432414	1.648957	0.035565	0.074602

### Identification of Prognosis-Related Genes

By univariate Cox regression analysis and Kaplan–Meier (KM) survival analysis (patients were classified into low and high expression groups based on median gene expression data), a total of 51 genes were screened out, of which 49 genes were unfavorable to prognosis (HR>1) and 2 genes were favorable to prognosis (HR<1) (**Table 3**).

**Table 3:** The prognostic related gene list.

Gene	KM	HR	HR.95L	HR.95H	coxPvalue
SLC38A1	0.003165	1.037392	1.014199	1.061115	0.001462
TXNL4A	0.001014	1.017909	1.006116	1.02984	0.002832
PGK1	0.000866	1.00361	1.00133	1.005895	0.001899
SORBS2	6.72E-05	0.969257	0.945723	0.993376	0.012776
PHB	0.002315	1.010017	1.003383	1.016693	0.00303
STIP1	0.00013	1.023625	1.015808	1.031503	2.37E-09
SNRPB2	0.000277	1.042278	1.021676	1.063296	4.80E-05
AMD1	1.14E-05	1.071158	1.042441	1.100666	7.13E-07
GAPDH	1.52E-05	1.00039	1.000175	1.000604	0.000365
CKS1B	0.00224	1.011813	1.004784	1.018892	0.000961
CCT7	0.000401	1.014353	1.008173	1.020571	4.86E-06
PSMA3	0.003753	1.01925	1.008159	1.030463	0.000636
CDC25B	0.002845	1.023348	1.011816	1.03501	6.56E-05
TPI1	0.000173	1.001565	1.000473	1.002658	0.004961
EIF2S1	0.003714	1.132416	1.08095	1.186332	1.61E-07
VDAC1	0.005192	1.007353	1.002668	1.012061	0.002071
ASNS	0.003391	1.02885	1.006893	1.051285	0.009763
EIF2S2	0.000218	1.023907	1.011755	1.036205	0.000105
SEC61A1	4.58E-06	1.008706	1.004549	1.012879	3.88E-05
CDK4	5.22E-05	1.018012	1.010166	1.02592	6.13E-06
AK2	0.005891	1.038733	1.01649	1.061463	0.00058
YKT6	0.000413	1.018644	1.00684	1.030588	0.001896
CDC42EP2	0.000296	1.091183	1.034936	1.150487	0.001231
NME1	0.001137	1.004218	1.00077	1.007676	0.016442
PSMD13	0.000882	1.017424	1.006653	1.02831	0.001467
CDCA8	2.39E-06	1.123353	1.085664	1.16235	2.38E-11
CDC20	3.85E-06	1.027371	1.01811	1.036715	5.07E-09
SNRPD1	0.000174	1.026441	1.010183	1.042961	0.001357
RAN	2.09E-05	1.010095	1.006302	1.013904	1.68E-07
SNRPA	8.27E-05	1.014106	1.005375	1.022913	0.001499
E2F2	5.30E-05	1.684961	1.322284	2.147112	2.45E-05
EFNA5	0.001057	1.082642	1.013996	1.155935	0.01751
ERH	0.000486	1.012517	1.005591	1.019491	0.000383
PPIA	0.000415	1.00327	1.001701	1.00484	4.32E-05
LDHA	8.70E-05	1.003787	1.002332	1.005243	3.25E-07
SRM	0.001693	1.010772	1.004765	1.016814	0.000427
DTYMK	1.32E-05	1.032033	1.017913	1.04635	7.26E-06
POLE4	0.018558	1.016728	1.004422	1.029185	0.007581
NT5C	0.001771	1.009348	1.000365	1.018411	0.041354
RBX1	0.006273	1.021113	1.005411	1.037061	0.008231
GRM8	0.000359	1.228372	1.108225	1.361545	8.98E-05
ADAD1	0.001756	1.77152	1.102922	2.845424	0.018023
RANBP1	7.04E-05	1.01552	1.008045	1.023051	4.40E-05
SORD	0.002689	0.993698	0.989909	0.997501	0.001178
PTGES3	2.89E-05	1.009964	1.004996	1.014956	8.11E-05
RAE1	0.000744	1.115734	1.066832	1.166877	1.68E-06
TBCD	0.000116	1.056685	1.018552	1.096244	0.00328
PSMA7	0.001897	1.004442	1.001317	1.007576	0.005301
EEF1E1	6.14E-07	1.045183	1.016904	1.074247	0.00159
CTSC	0.000925	1.005762	1.001276	1.010267	0.011757
POLR2G	0.001017	1.023782	1.011387	1.03633	0.000156

### Construction of a Six-Gene Prognostication Signal in the Training Cohort

**Figure 3:**
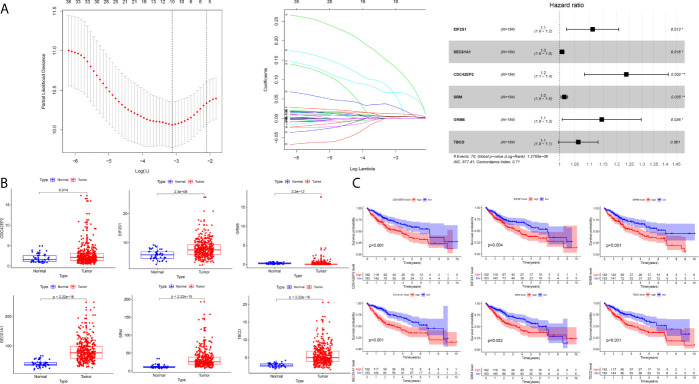
Construction of the prognostic model in the training cohort **(A)** The building process of the six-gene prognostic signature **(B)** The expression level of the six genes between tumor and normal tissues **(C)** The Kaplan–Meier survival curve of the expression level of the six genes and overall survival.

**Figure 4:**
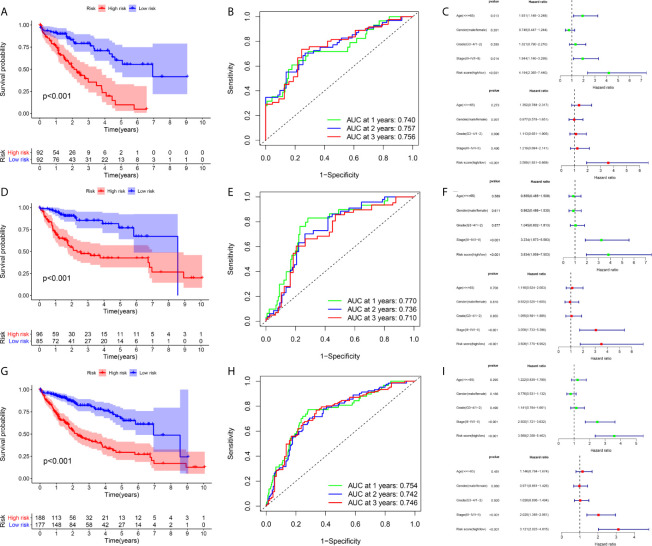
Prognostic assessment of the prognostic model in the TCGA cohort **(A–C)** The Kaplan–Meier survival analysis, time−dependent ROC analysis, and independence analysis of the signature for predicting the overall survival of patients in the training cohort (n=184) **(D–F)** The Kaplan–Meier survival analysis, time−dependent ROC analysis, and independence analysis of the signature for predicting the overall survival of patients in the testing cohort(n=181) **(G–I)** The Kaplan–Meier survival analysis, time−dependent ROC analysis, and independence analysis of the signature for predicting the overall survival of patients in the entire TCGA cohort(n=365). Green represents univariate analysis, and red represents multivariate analysis.

A total of 184 HCC patients were enrolled in the training set. The flowchart of the prognosis-scoring model construction process is shown in **Figure 3A**. The aforementioned 51 genes were further reduced by Lasso penalty Cox regression analysis, and we subsampled the dataset 1000 times with 10-fold cross-validation. After that, 41 genes with zero Lasso regression coefficients were excluded with the optimal value of log λ (- 3.2), and 10 genes with nonzero Lasso regression coefficients were included in the multivariate Cox regression analysis. A novel risk score was calculated by multiplying the gene expression of each gene and its corresponding coefficient, which was obtained by multivariate Cox regression analysis. Risk score= EIF2S1*0.1055 + SEC61A1* 0.0082 + CDC42EP2*0.2127 + SRM*0.0155 + GRM8*0.1344 + TBCD*0.0600. The patients were grouped into high-risk or low-risk categories using the median risk score of the training series as the cutoff point (0.81). There was a significant difference in RNA expression level between tumor (n = 374) and normal tissues (n = 50) of the 6 genes in the signature (**Figure 3B**). Higher levels of the six mRNAs were associated with decreased overall survival (OS) in the TCGA cohort (n = 365) (**Figure 3C**). The OS of HCC patients in the cohort exhibiting high risk was significantly lower than that of the cohort exhibiting low risk (**Figure 4A**, P < 0.001). ROC curve analysis demonstrated the predictive ability of the risk score for 1-, 2- and 3-year OS, with areas under the curve (AUCs) of 0.740, 0.757 and 0.756, respectively (**Figure 4B**). Univariate and multivariate Cox regression analyses showed that only the risk score of the 6-gene signature was an independent prognostic element (**Figure 4C**).

### Internal Validation of the Prognostic Signature in the Testing and Entire TCGA Cohort

An identical risk score formula and cutoff value determined from the training set were used to analyze the testing cohort. Consistent with the results in the training set, the KM curves of the testing sets showed that the cohort exhibiting high risk had a worse prognosis than the low-risk group (**Figure 4D**). The AUC values for the signature predicting OS at 1, 2 and 3 years were 0.770, 0.736 and 0.710, respectively (**Figure 4E**), indicating good prediction accuracy. From the results of both univariate and multivariate Cox regression analyses, the risk score was found to be an independent poor prognostic indicator of HCC (**Figure 4F**). To validate the accuracy of the risk model, we analyzed the model in the entire TCGA cohort, and the results were in line with the above-described results (**Figures 4G–I**).

### External Validation of the Prognostic Signature in the ICGC Cohort

**Figure 5:**
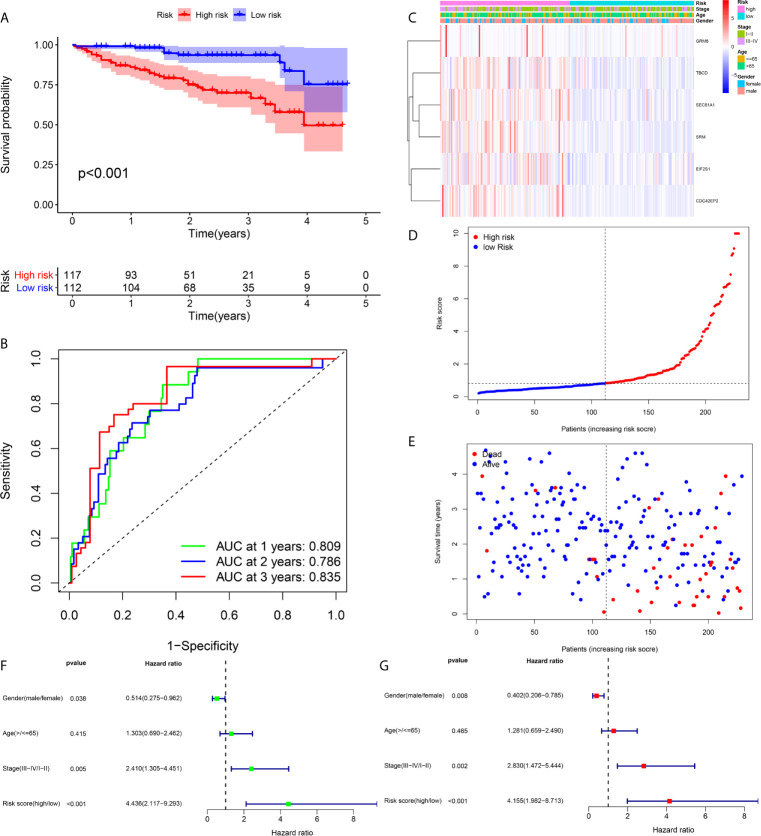
Prognostic assessment of the prognostic model in the ICGC cohort **(A, B)** The Kaplan–Meier survival analysis and time−dependent ROC analysis of the signature for predicting the overall survival of patients in the ICGC cohort (n = 229) **(C)** The heatmap of the six-gene signature **(D)** The survival status of patients **(E)** The distribution of the risk score **(F, G)** Univariate and multivariate regression analyses of the relation between the risk score and clinicopathological characteristics regarding the overall survival in the ICGC cohort (green represents univariate analysis, and red represents multivariate analysis).

For the in-depth assessment of the reliability of the prognostication model, an external dataset from the ICGC database was employed to verify the six-gene signature. The risk score was determined for each case according to the six-gene signature, and the 229 patients were divided into a high- or low-risk cohort based on their risk score. The Kaplan–Meier plot indicated that patients in the cohort exhibiting high risk had significantly shorter overall survival than those in the low-risk group (**Figure 5A**). The AUCs at 1, 2 and 3 years were 0.835, 0.786, and 0.809, respectively, indicating that the risk score played a significant role in the prediction of prognosis (**Figure 5B**). As shown in **Figure 5C**, the six-gene expression level was increased significantly in the high-risk group. The risk of mortality tended to rise along with the risk score (**Figures 5D, E**). Both univariate and multivariate analyses suggested that the risk score could be used as an independent prognostic indicator (**Figures 5F, G**).

### Gene Set Variation Analysis Between Different Risk Groups

**Figure 6:**
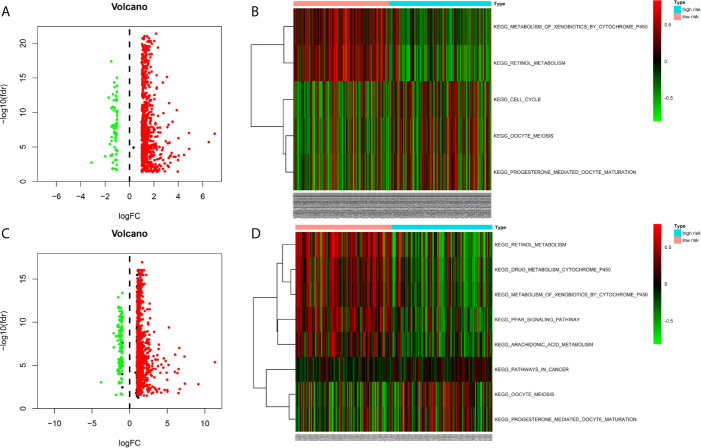
Gene Set Variation Analysis between high- and low risk groups in the TCGA and ICGC cohort **(A)** Differentially expressed genes (DEGs) between high- and low-risk groups in the TCGA cohort **(B)** Gene Set Variation Analysis between high- and low risk groups in the TCGA cohort **(C)** Differentially expressed genes (DEGs) between high- and low-risk groups in the ICGC cohort **(D)** Gene Set Variation Analysis between high- and low risk groups in the ICGC cohort.

We identified the differentially expressed genes (DEGs) between the high‐risk and low‐risk groups in the ICGC and TCGA datasets (**Figures 6A, C**). Subsequently, we made use of these DEGs to perform gene set variation analysis (GSVA). KEGG pathway activities were scored per sample by GSVA between the different risk groups. We found a significant decrease in metabolism-related signaling pathway GSVA scores in the cohort exhibiting high risk (**Tables 4**, **5** and **Figures 6B, D**). Furthermore, oocyte meiosis-associated genes exhibited higher enrichment scores in the cohort demonstrating high risk (**Tables 4**, **5** and **Figures 6B, D**).

**Table 4:** Gene Set Variation Analysis between high- and low risk groups in the TCGA cohort.

NAME	logFC	AveExpr	t	P.Value	adj.P.Val	B
KEGG_METABOLISM_OF_XENOBIOTICS_BY_CYTOCHROME_P450	0.252025	0.049534	9.814887	2.15E-20	3.44E-19	35.49329
KEGG_RETINOL_METABOLISM	0.287397	0.033523	9.701779	5.24E-20	7.85E-19	34.61556
KEGG_OOCYTE_MEIOSIS	-0.12728	-0.02586	-3.74371	0.00021	0.002936	-0.4066
KEGG_CELL_CYCLE	-0.11486	-0.03202	-3.45642	0.00061	0.00793	-1.40409
KEGG_PROGESTERONE_MEDIATED_OOCYTE_MATURATION	-0.10682	-0.01851	-3.00679	0.002818	0.033812	-2.81452

**Table 5:** Gene Set Variation Analysis between high- and low risk groups in the ICGC cohort.

NAME	logFC	AveExpr	t	P.Value	adj.P.Val	B
KEGG_RETINOL_METABOLISM	0.330529	0.056794	8.797863	2.89E-16	5.19E-15	26.25868
KEGG_PPAR_SIGNALING_PATHWAY	0.242533	0.034012	7.812051	1.80E-13	3.05E-12	19.93605
KEGG_DRUG_METABOLISM_CYTOCHROME_P450	0.248736	0.027323	7.477833	1.44E-12	2.31E-11	17.89126
KEGG_METABOLISM_OF_XENOBIOTICS_BY_CYTOCHROME_P450	0.234159	0.029967	7.336734	3.43E-12	5.14E-11	17.0445
KEGG_ARACHIDONIC_ACID_METABOLISM	0.166018	-0.00097	5.454549	1.23E-07	1.72E-06	6.826222
KEGG_CHEMOKINE_SIGNALING_PATHWAY	0.087317	0.061533	3.573573	0.000426	0.005541	-0.95374
KEGG_OOCYTE_MEIOSIS	-0.12687	-0.04236	-3.35872	0.000912	0.010941	-1.66122
KEGG_PROGESTERONE_MEDIATED_OOCYTE_MATURATION	-0.12095	-0.0516	-3.26597	0.001252	0.013767	-1.9543
KEGG_PATHWAYS_IN_CANCER	-0.05659	-0.01159	-3.0279	0.002734	0.027337	-2.67212

### Differences in the Immune Landscape Between Different Risk Groups

**Figure 7:**
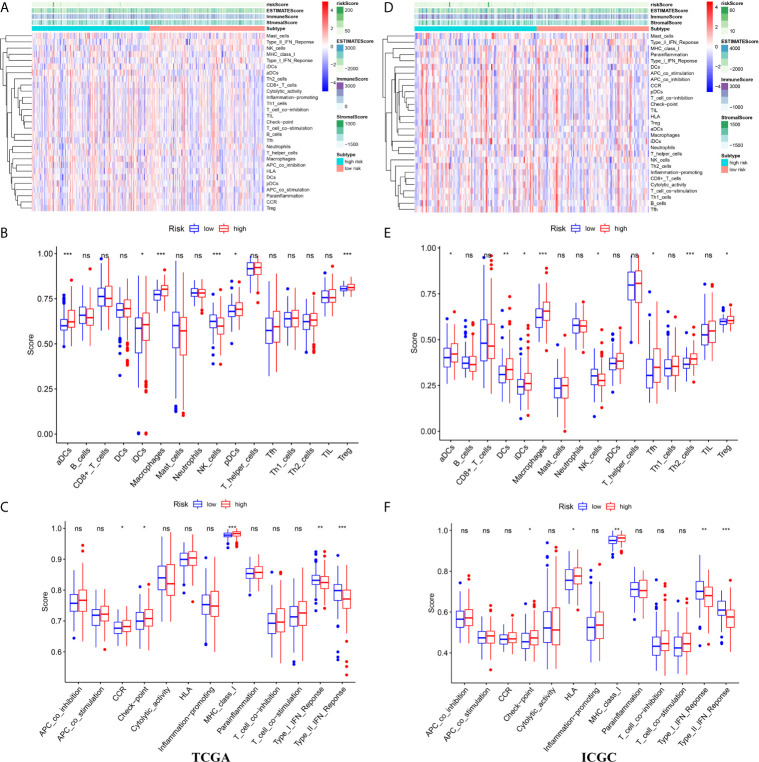
The immune landscape between high- and low risk groups in the TCGA and ICGC cohort **(A)** The heatmap of immune cell infiltration and immune function in the TCGA cohort **(B)** The differences of immune cell infiltration between high- and low risk groups in the TCGA cohort **(C)** The differences of immune function between high- and low risk groups in the TCGA cohort **(D)** The heatmap of immune cell infiltration and immune function in the ICGC cohort **(E)** The differences of immune cell infiltration between high- and low risk groups in the ICGC cohort **(F)** The differences of immune function between high- and low risk groups in the ICGC cohort. ***p < 0.001; **p < 0.01; *p < 0.05. ns, no significant.

**Figure 8:**
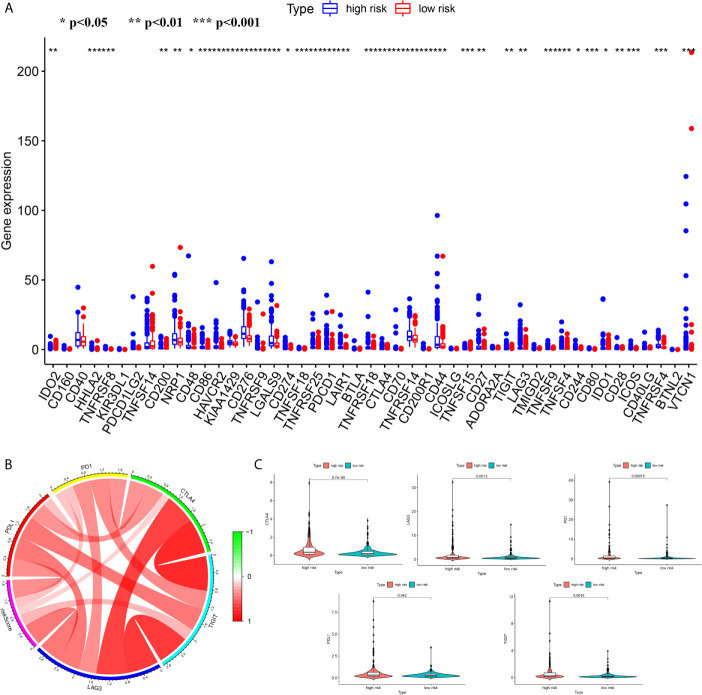
The differences of the expression level of immune checkpoint between high- and low risk groups **(A)** The boxplot of the expression level of immune checkpoint between high- and low risk groups **(B)** The correlation analysis between the risk score and the expression levels of PD1, PDL1, TIGIT, CTLA4 and LAG3 **(B, C)** The Comparison of the expression level of PD1, PDL1, TIGIT, CTLA4 and LAG3 between high- and low risk groups. ***p < 0.001; **p < 0.01; *p < 0.05.

We attempted to explore cases involving differences in prognosis in different risk groups from the perspective of the immune microenvironment. We used the ssGSEA score to quantify the activity or enrichment levels of immune cells and functions in the HCC samples (**Figures 7A, D**). Similar results were obtained from two independent cohorts. In terms of immune cell infiltration, the infiltration abundance of aDCs, iDCs, macrophages, NK cells, and Tregs in the cohort exhibiting high risk was significantly higher than that in the low-risk group (**Figures 7B, E**). In terms of immune function, the results revealed that the expression of immune checkpoints and MHC class I-related gene sets was upregulated in the high-risk group, while the expression of type I and II IFN response-related gene sets was upregulated in the cohort exhibiting low risk (**Figures 7C, F**). We also detected the expression levels of PD1 (PDCD1), PDL1, TIGIT, CTLA4 and LAG3 in the high-risk and low-risk groups. This study reported that the risk score positively correlated with the expression levels of PD1 (PDCD1), PDL1, TIGIT, CTLA4 and LAG3. The expression levels of these five common immune checkpoints were all upregulated in the high-risk cohort (**Figures 8A–C**).

### Integrated Analysis of the Prognostication Signal in the Whole Group

**Figure 9:**
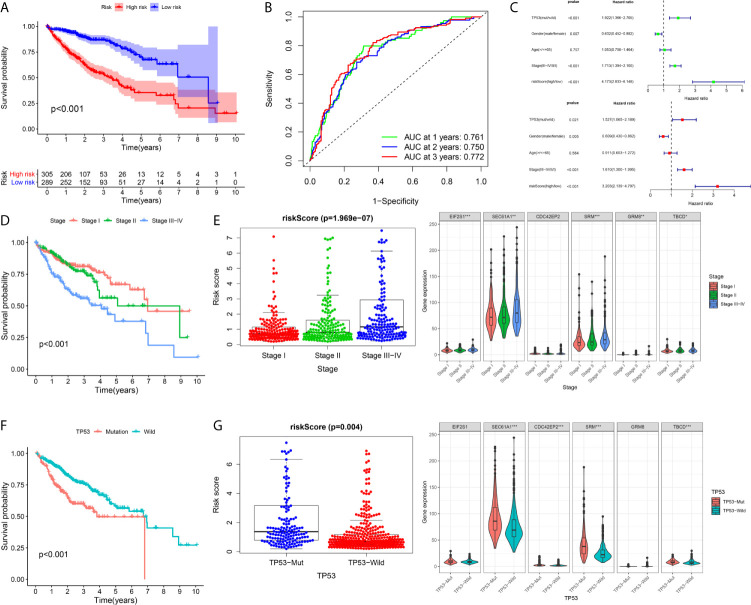
Integrated analysis of the prognostic signature in whole cohort **(A–C)** The Kaplan–Meier survival analysis, time−dependent ROC analysis, and independence analysis of the signature for predicting the overall survival of patients in the whole cohort(n=594) (green represents univariate analysis, and red represents multivariate analysis) **(D, E)** The relationship between the risk score and TNM-stage **(F, G)** The relationship between the risk score and TP53. ***p < 0.001; **p < 0.01; *p < 0.05.

A total of 594 cases were covered in this pooled analysis. The results were consistent with the conclusion from a previous study. In terms of OS, patients exhibiting high risk had a poorer OS than patients exhibiting low risk (**Figure 9A**). The AUC values of the 1-year, 2-year and 3-year overall survival rates of patients predicted by the risk score were 0.761, 0.750, and 0.772, respectively (**Figure 9B**). Univariate and multivariate Cox regression analyses revealed that a high risk score indicated poor clinical outcome in HCC patients (**Figure 9C**). In addition, TP53 and TNM stage were significant prognostic elements (**Figures 9D, F**), and a positive correlation was found between risk score and TNM stage (**Figure 9E**). Patients with TP53 mutations had higher risk scores (**Figure 9G**).

### Subgroup Survival Analysis

**Figure 10:**
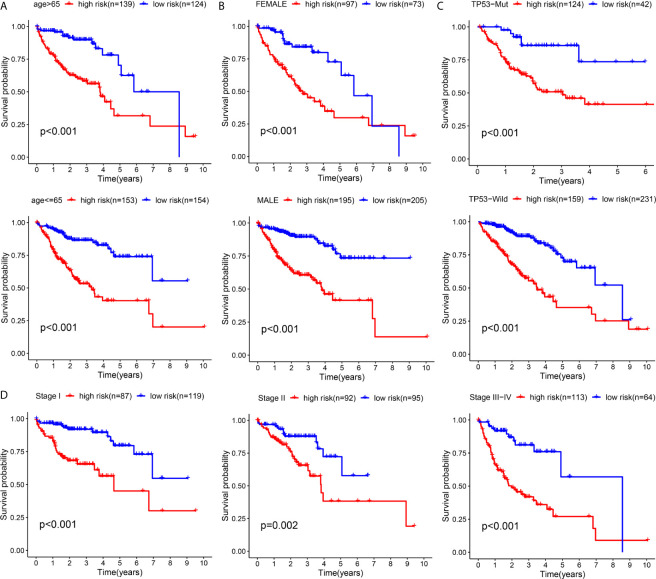
Subgroup Kaplan–Meier survival analysis according to different clinical features in all included HCC patients **(A)** Age **(B)** Gender **(C)** TP53 **(D)** TNM-stage.

To validate the general applicability of the prognostication signal in HCC, we grouped the 594 patients according to their clinical characteristics. In each subgroup, cases exhibiting high risk were compared with those exhibiting low risk. The prognostication of cases with high risk was noticeably worse than that of cases with low risk in each subgroup (**Figures 10A–D**). These results confirmed that the risk score had good stratification ability for the prognosis of HCC.

## Discussion

A majority of HCC patients are at the medium and advanced phases when diagnosed because there are no obvious symptoms in the early stage of HCC (18). Although many therapeutic strategies have been attempted, such as radiofrequency ablation, surgical resection and liver transplantation, the prognostication of HCC cases receiving these treatments is still unsatisfactory (19). Elucidating the molecular mechanism and process of HCC is very important for identifying new therapeutic targets and improving the clinical outcomes of HCC patients.

At present, the traditional methods for predicting prognosis based on a single biomarker or clinical data lack systematic evaluation, resulting in a lack of high sensitivity and specificity. Due to the complex molecular regulation mechanism of HCC, traditional clinical pathological staging cannot fully reflect the heterogeneity of tumors, and the ability to predict prognosis is poor. Therefore, the construction of a gene sequencing prediction model based on big data has great potential for clinical transformation and has become an urgent need to improve clinical efficacy.

The TP53 mutation process is one of the most common mutations in HCC and is considered to be the main driving factor of HCC (8, 18). This gene plays a vital role in maintaining genomic stability, and its loss of function can lead to centrosome amplification, aneuploid cell proliferation and chromosome instability (20). Compared with TP53 wild-type HCC patients, TP53 mutant HCC patients had shorter overall survival and relapse-free survival (13). We speculated that the poor prognosis of HCC patients with TP53 mutations may be partly due to the change in the RNA expression pattern caused by TP53 mutation. Following this hypothesis, we performed GSEA on HCC samples with and without TP53 mutation. We identified 7 gene sets that were positively correlated with TP53 mutation. After univariate Cox regression analysis, Kaplan–Meier (KM) survival analysis, Lasso penalty Cox regression analysis, and step-by-step multivariate Cox regression analysis, we selected six genes (EIF2S1, SEC61A1, CDC42EP2, SRM, GRM8, TBCD) to construct a prognostic risk score model.

To assess the reliability of the prognostic model, we conducted internal validation, external validation, integrated analysis and subgroup analysis. The AUC values of the ROC curves of 1-, 2-, and 3-year survival of the model were all greater than 0.7 in each independent cohort, which indicated that the signature composed of six genes had good performance in predicting the prognosis of HCC. The proposed signature was superior to a previously TP53-associated prognostic model developed by Long et al. (21) for the assessment of HCC OS.

To explore the regulatory mechanisms of the prognostication model, we performed GSVA on different risk groups. This work indicated that the high-risk cohort exhibited stronger proliferative activity and weaker metabolic activity, which was similar to the results of Gao et al. (22). Based on analysis of the tumor immune microenvironment, the cohort exhibiting high risk showed strong immunosuppression. Because of several important components representing immunosuppression, such as macrophages and Tregs (23, 24), the infiltration abundance in the high-risk group was significantly higher than that in the low-risk group. Another important result was that the expression levels of immune checkpoints in the cohort with high risk were significantly higher than those in the cohort with low risk, which was consistent with the positive correlation between the risk score and the expression level of immune checkpoints. Interestingly, the risk score corresponding to TP53 mutation was also significantly increased. As suggested by Long et al. (25), the immune response of TP53 mutant HCC was significantly weaker. Therefore, we speculated that the poor prognosis of HCC patients in the cohort exhibiting high risk was associated with the tumor microenvironment of immunosuppression. Based on this evidence, we summarized the possible reasons that may lead to weak prognosis in the high-risk group: high proliferative activity, low metabolic activity and immunosuppression.

To date, the six genes in the signature have not been reported in HCC. EIF2S1 participates in premature protein synthesis processes through the formation of a ternary complex with initiators tRNA and GTP (26). SEC61A1 refers to the main polypeptide conduction pathway in the endoplasmic reticulum membrane and the main subunit of the SEC61 complex. Its missense mutation can cause genetic immune-related diseases, such as plasma cell deficiency (27). CDC42EP2 is a member of the CDC42 subfamily that belongs to the Rho family, and the Rho family plays an important role in a variety of cellular processes covering skeletal myogenesis (28). SRM refers to a polyamine biosynthetic enzyme (29). Polyamines modulate the gene expression process through changes in DNA structures and the regulation of signal transduction pathways, and they are consequently associated with proliferation, tumor invasion, and metastasis (30, 31). GRM8 pertains to 1 of eight G-protein coupled receptors in the glutamate family and couples to various intracellular second messenger mechanisms to modulate neuronal functions (e.g., neuronal excitability and development) (32). TBCD encodes TBCD (tubulin folding cofactor D), one of five tubulin-specific chaperones that critically impact microtubule assembly in all cells (33). Recently, variants in TBCD have been identified in patients with distinct progressive encephalopathy with an apparently wide clinic-related scope (34).

Our study used TP53 to find 6 new prognostic biomarkers of HCC, and the signature composed of these six genes showed good performance in predicting the prognosis of HCC. However, this work had limits that should be acknowledged. We developed and validated the prognostic risk mode by utilizing general databases, and the outcomes require in-depth confirmation through prospective research. Our major findings were generated from bioinformatics analysis, which demonstrates insufficient verification through *in vitro* and *in vivo* experimental processes. In future work, prospective laboratory studies to clarify the specific mechanisms of the six genes in our signature are warranted.

## Conclusion

Our study provides a reliable method for the prognostic risk assessment of HCC and has great potential for clinical transformation.

## Data Availability Statement

Publicly available datasets were analyzed in this study. This data can be found here: The datasets analysed for this study were obtained from The Cancer Genome Atlas (TCGA) (https://portal.gdc.cancer.gov/) and International Cancer Genome Consortium (ICGC) (https://icgc.org/).

## Author Contributions

JH designed this study. LW and YZ contributed to the conception of the study. JH performed the data analyses and wrote the manuscript. LW helped perform the analysis with constructive discussions. All authors contributed to the article and approved the submitted version.

## Conflict of Interest

The authors declare that the research was conducted in the absence of any commercial or financial relationships that could be construed as a potential conflict of interest.

## Abbreviations

HCC, hepatocellular carcinoma; TCGA, The Cancer Genome Atlas; ICGC, International Cancer Genome Consortium; GSEA, Gene Set Enrichment Analysis; KM, Kaplan–Meier; DEGs, differential expressed genes; GSVA, Gene Set Variation Analysis; ssGSEA, single sample gene set enrichment analysis; KEGG, Kyoto Encyclopedia of Genes and Genomes; ROC, receiver operating characteristic; AUC, area under the curve; OS, overall survival; LASSO, Least absolute shrinkage and selection operator; FDR, false discovery rate.
